# Retinal Photoreceptor Protection in an AMD-Related Mouse Model by Selective Sigma-1 or Sigma-2 Receptor Modulation

**DOI:** 10.3390/genes13122386

**Published:** 2022-12-16

**Authors:** Timur A. Mavlyutov, Jing Li, Xinying Liu, Hongtao Shen, Huan Yang, Christopher R. McCurdy, Bikash Pattnaik, Lian-Wang Guo

**Affiliations:** 1 Department of Surgery, University of Wisconsin, Madison, WI 53705, USA; 2 Department of Surgery, School of Medicine, University of Virginia, Charlottesville, VA 22908, USA; 3 Department of Pediatrics, University of Wisconsin, Madison, WI 53705, USA; 4 Department of Medicinal Chemistry, College of Pharmacy, University of Florida, Gainesville, FL 32610, USA; 5 Department of Ophthalmology and Visual Sciences, University of Wisconsin, Madison, WI 53705, USA; 6 McPherson Eye Research Institute, University of Wisconsin, Madison, WI 53705, USA; 7 Department of Ophthalmology, School of Medicine, University of Virginia, Charlottesville, VA 22908, USA

**Keywords:** sigma-1 and sigma-2 receptors, selective ligands, photoreceptor protection, AMD-related model, retinal pigment epithelium

## Abstract

The structurally and genetically distinct sigma-1 receptor (S1R) and sigma-2 receptor (S2R) comprise a unique class of drug binding sites. Their alleles are associated with human diseases involving neuronal systems, such as age-related macular degeneration (AMD) characterized by photoreceptor and retinal pigment epithelium (RPE) atrophy. Previous studies have suggested neuroprotective benefits for the brain and retina from pharmacological modulation of S1R and/or S2R. However, the effect of such modulation on AMD pathology remains underexplored. Here, we evaluated S1R- or S2R-selective modulation in an AMD-related model of *Abca4*−/−*Rdh8*−/− mice with a disrupted visual cycle that predisposes RPE and photoreceptors to illumination-induced damage. For S1R modulation, we used (+)-pentazocine, which is a high-affinity S1R-selective drug. For S2R modulation, we chose CM398, a high-affinity and highly S2R-selective ligand with drug-like properties. *Abca4*−/−*Rdh8*−/− mice received a single i.p. injection of (+)-pentazocine or CM398 or vehicle 30 min before illumination. Pretreatment with (+)-pentazocine improved electroretinogram a- and b-waves compared to that with vehicle. Consistently, in another AMD-related mouse model induced by tail-vein injected NaIO_3_, S1R genetic ablation aggravated photoreceptor loss. In *Abca4*−/−*Rdh8*−/− mice, pretreatment with CM398 appeared to partially avert illumination-induced photoreceptor loss and autofluorescent granule formation that signals RPE damage, as revealed by optical coherence tomography. Thus, this study using AMD-related models provides evidence of photoreceptor protection afforded by selective modulation of S1R or S2R.

## 1. Introduction

The sigma-1 receptor (S1R) and the sigma-2 receptor (S2R) are intriguing drug targets. They were initially categorized as opioid receptors [1] but later discovered to be a unique class of non-opioid receptors [2,3] that are encoded by two different genes [4,5]. Many small molecule ligands bind to both S1R and S2R [6,7], yet recent reports revealed distinct ligand-binding structures in these two receptors [8,9,10]. Enormous medicinal chemistry efforts have been dedicated to improving ligand selectivity between S1R and S2R and reducing off-target affinities. This has been matched with increased efforts in preclinical and clinical therapeutic studies. In ongoing clinical trials, S1R and/or S2R are targeted to treat neuropathic pain, Alzheimer’s disease, schizophrenia, ischemic stroke, and COVID-19 [11].

Alleles of the S1R and S2R genes have been associated with neural system diseases such as amyotrophic lateral sclerosis [12] and age-related macular degeneration (AMD) [13,14]. Based on the literature, activation of S1R with agonists appears to be neuroprotective in general [15,16]. However, the specific biological and molecular functions of S2R are still poorly understood, likely because it was only recently found to be coded by the TMEM97 gene [5]. Although the role of S2R/TMEM97 in the retina has been underexplored [17], there is evidence that S2R modulators could be neuroprotective for the brain [11,18] and/or retinal ganglion cells [19]. In an oxidant-induced retinal degeneration model, we observed that photoreceptor loss was exacerbated in *Tmem97*−/− mice compared to *Tmem97*+/+ mice [17]. However, there is a lack of published work on S2R-selective pharmacological modulation in an AMD-related animal model involving both photoreceptor neurons and the retinal pigment epithelium (RPE).

Studies on the role of S1R in the retina have focused on neurons, mainly photoreceptors and ganglion cells [16,20]. For example, the Smith group has made a series of findings of photoreceptor protection through S1R pharmacological activation in mouse models of retinitis pigmentosa (RP) or diabetic retinopathy [21,22,23,24]. Whether targeting S1R pharmacologically or genetically in an AMD-related model influences retinal degeneration remains largely unknown.

RPE is a non-neuronal tissue derived from the neuroectoderm and underlies photoreceptor neurons to critically support their function and survival [25]. RPE dysfunction is etiological to AMD [26]. The Palczewski group developed a retinal illumination model with *Abca4*−/−*Rdh8*−/− mice featuring a disrupted visual cycle that is otherwise formed between photoreceptor and RPE cells [27]. Since this model has shown promise in recapitulating salient characteristics of dry AMD [27,28], we used the same model in the current study. We performed live animal characterizations, including recording of electroretinograms (ERGs) and optical coherence tomography (OCT). Our objective was to gain evidence as to whether selectively targeting S1R or S2R in AMD-related mouse models could result in photoreceptor protection.

## 2. Results

### 2.1. S1R-Selective Modulation with (+)-Pentazocine in Abca4−/−Rdh8−/− Mice Protects Photoreceptors from Illumination-Induced Damage

**Figure 1:**
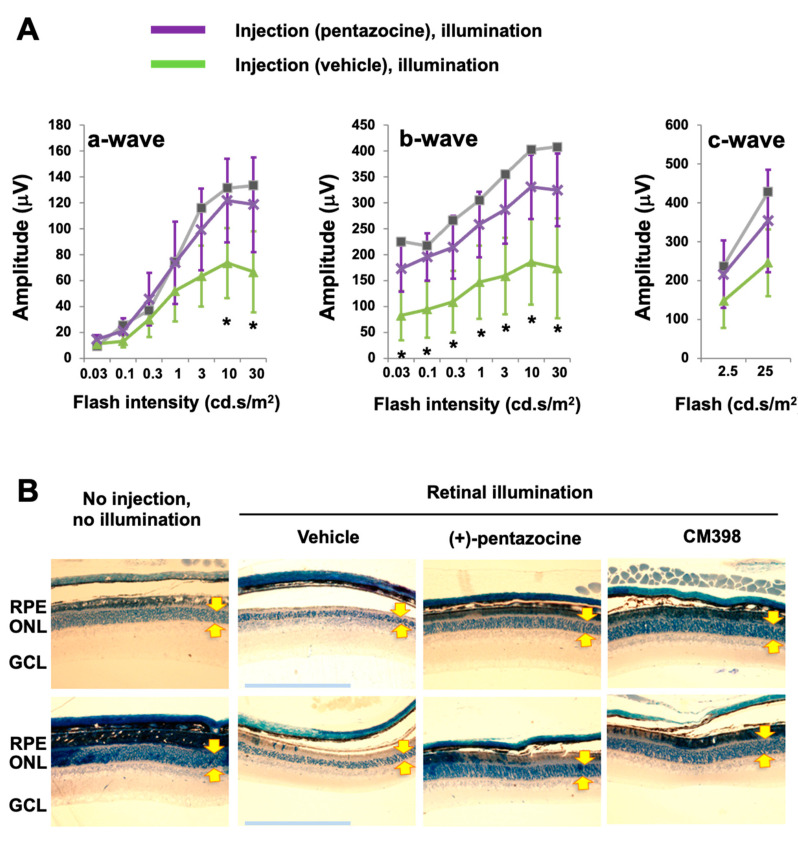
Pretreatment with (+)-pentazocine protects photoreceptor function in *Abca4*−/−*Rdh8*−/− mice. Mice received a one-time i.p. injection of 1 mg/kg (+)-pentazocine 30 min before retinal illumination (10,000 lux for 30 min). The mice were subjected to ERG recording 7 days after housing in the dark (**A**), and then euthanized for retinal histology (**B**). (**A**) ERGs. Student’s *t*-test was performed to compare the (+)-pentazocine-treated group and the vehicle control group at each scotopic light intensity, mean ± SD, *n* = 4 mice in each group, * *p* < 0.05. The gray curve measured with one mouse represents the basal condition (no injection, no illumination). (**B**) Methylene blue-stained retinal sections. Images shown are representative sections from two animals for each treatment group. The imaged region is within 1000–2000 μm from the optic nerve head. Arrows demarcate ONL thickness. RPE: retinal pigment epithelium. ONL: outer nuclear layer. GCL: ganglion cell layer. Scale bar: 200 μm.

In this study, we chose (+)-pentazocine for S1R modulation for two main reasons: (1) it is well-established as a high-affinity (6.7 nM), high-selectivity S1R ligand (>200 fold over S2R) [6] and (2) it has been repeatedly shown to be photoreceptor-protective in RP and diabetic retinopathy models [21,22,23,24] but has not been tested in an AMD-related model in vivo [16]. We thus used (+)-pentazocine to eliminate the choice of S1R modulator as an experimental variable. Moreover, this established S1R agonist appeared to be ideal as a “model drug” for us to validate our experimental setting of retinal illumination in *Abca4*−/−*Rdh8*−/− mice related to AMD pathology. We performed a single, one-time i.p. injection of (+)-pentazocine (1 mg/kg) in *Abca4*−/−*Rdh8*−/− mice 30 min before retinal illumination (10,000 lux for 30 min), and recorded ERGs 7 days later. While a-waves reflect phototransduction responses in rod photoreceptors, b-waves reflect the activities of inner retinal neurons, especially bipolar cells but are strongly influenced by photoreceptor function, and c-waves are often used to monitor the function of the RPE [29]. As indicated in Figure 1, in the mice treated with (+)-pentazocine compared to the vehicle control group, the a-wave amplitude increased at the light intensities of 10 and 30 cd·s/m^2^ and the b-wave amplitude increased at all light intensities. The c-wave showed a trend of improvement, albeit without reaching statistical significance. This result was also reflected in the stained retinal sections where the thinning of the outer nuclear layer (ONL) due to retinal illumination appeared to be partially rescued by pretreatment of the mice with (+)-pentazocine (Figure 1B).

Thus, S1R modulation with (+)-pentazocine was protective against illumination-induced photoreceptor dysfunction as detected by ERG in *Abca4*−/−*Rdh8*−/− mice.

### 2.2. S1R Ablation Exacerbates Oxidant-Induced Retinal Degeneration

**Figure 2:**
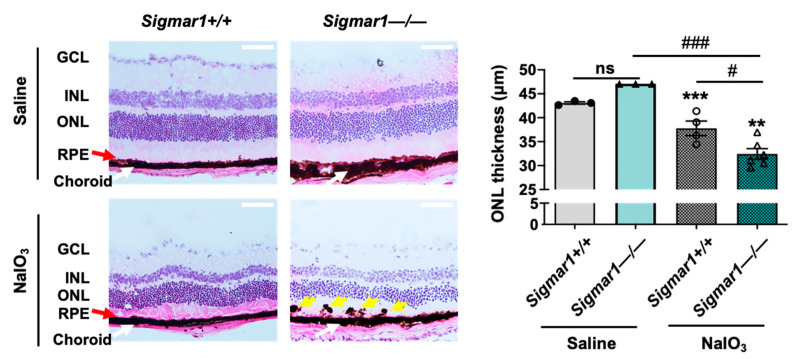
Comparison of photoreceptor loss in NaIO_3_-challenged *Sigmar1*+/+ and *Sigmar1*−/− mice. Mice received saline (PBS) or 30 mg/kg NaIO_3_ through tail-vein injection. Eyeballs were collected 3 days after injection, and fixed retinal sections were H&E stained. RPE: retinal pigment epithelium. ONL/INL: outer/inner nuclear layer. GCL: ganglion cell layer. Red arrow marks the RPE layer. Yellow arrow points to pigmented blebs in the RPE layer. Scale bar: 50 μm. Quantification: Data are presented as mean ± SEM. In the NaIO_3_-treated group, *n* = 4 *Sigmar1*+/+ mice (8 eyes) or *n* = 6 *Sigmar1*−/− mice (12 eyes); in the saline-treated group, *n* = 3 *Sigmar1*+/+ mice (6 eyes) or 3 *Sigmar1*−/− mice (6 eyes). Statistics: one-way ANOVA and Tukey test; # *p* < 0.05, ### *p* < 0.001; ns, not significant. ** *p* < 0.01, *** *p* < 0.001, compared to the first bar.

To further determine the specific role of S1R in retinal degeneration in an AMD-related mouse model, we performed experiments using a *Sigmar1*−/− mouse line that has been established in previous reports [30,31]. To avoid lengthy breeding and complex selection required to generate a *Sigmar1*−/−*Abca4*−/−*Rdh8*−/− triple mutant mouse line, we performed tail-vein injection of NaIO_3_ in *Sigmar1*+/+ and *Sigmar1*−/− mice and determined the retinal ONL thickness three days after injection, by following the method in our recent report [17]. In this well-characterized AMD-related mouse model, the oxidant NaIO_3_ primarily damages the RPE ultimately leading to photoreceptor cell loss [32]. As seen in Figure 2, the ONL thickness was significantly lower in *Sigmar1*−/− mice than that in *Sigmar1*+/+ mice, which were both treated with NaIO_3_ for three days. There was no significant difference in ONL thickness between *Sigmar1*−/− and *Sigmar1*+/+ mice injected with saline instead of NaIO_3_. Thus, the result from S1R genetic ablation concurs with that from pharmacological modulation of S1R, further supporting a photoreceptor-protective role of S1R in AMD-related mouse models.

### 2.3. S2R-Selective Modulation with CM398 in Abca4−/−Rdh8−/− Mice Partially Rescues Photoreceptor Loss Caused by Retinal Illumination

**Figure 3:**
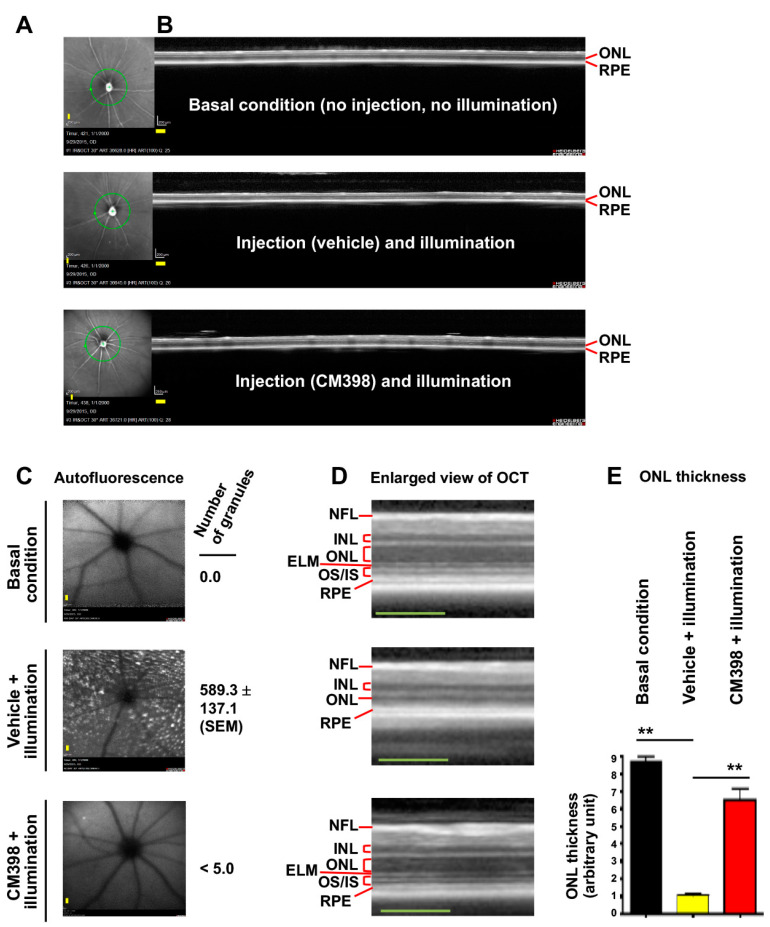
Effect of pretreatment with CM398 on photoreceptor loss induced by retinal illumination in *Abca4*−/−*Rdh8*−/− mice. Mice received a one-time i.p. injection of CM398 (3 mg/kg) 30 min prior to retinal illumination (10,000 lux for 30 min). Retinal OCTs were recorded after housing the mice in a room with red dim light for 7 days. The mice were then euthanized for retinal histology (see Figure 1B). Each scale bar (yellow) in the OCT images represents 200 μm. (**A**) Fundus photography. The green circle around the optic nerve head indicates the location of the vertical SD-OCT scan. (**B**) Full image of vertical SD-OCT scan. The scale (200 μm) and experiment date are recorded at the lower left corner. (**C**) Scanning laser ophthalmoscopy. The number of autofluorescence granules was quantified using Image-J. Mean ± SEM, *n* = 6 mouse eyes. (**D**) Enlarged view of the OCT image in B. RPE: retinal pigment epithelium. OS/IS: outer/inner segment. ELM: external limiting membrane. ONL: outer nuclear layer. INL: inner nuclear layer. NFL: nerve fiber layer. (**E**) Quantification for A. ONL thickness was measured using Image-J. The values from 3–4 OCT images of the same eye were averaged, and the means from all 6 eyes from 3 animals were then averaged to calculate each group’s mean and standard error of the mean (SEM). Data are presented as mean ± SEM, *n* = 6 mouse eyes. One-way ANOVA and Tukey test, ** *p* < 0.01.

To selectively target S2R, we chose CM398 for its superior affinity (0.43 nM) and selectivity for S2R (>1000-fold over S1R) [6,33] and its drug-like properties [34]. While CM398 is a putative S2R antagonist [6], another putative S2R antagonist CT1812, though less selective between S2R and S1R, has been reported to be neuroprotective in mice and is in human clinical trials for Alzheimer’s disease [35]. We administered a single, one-time i.p. injection of CM398 (3 mg/kg) 30 min before retinal illumination and performed optical coherence tomography (OCT) imaging on post-illumination day 7. As shown in Figure 3, whereas retinal illumination diminished the ONL, pretreatment with CM398 largely restored the thickness of this layer of photoreceptors compared to the basal condition group without injection and illumination. Moreover, pretreatment with CM398 abrogated illumination-induced accumulation of granular autofluorescence that is thought to be predominantly emitted by harmful metabolic wastes such as lipofuscin in RPE cells [28,36]. The stained retinal sections also illustrated the protective effect of CM398 (Figure 1B).

Thus, these results suggest that S2R modulation with CM398 partially rescues the illumination-induced phenotype of ONL loss and accumulation of retinal autofluorescent granules in *Abca4*−/−*Rdh8*−/− mice.

## 3. Discussion

S2R has been studied pharmacologically for decades, yet the effect of its selective ligands on photoreceptor survival remains largely unknown. The S1R-selective drug (+)-pentazocine has been reported to protect rod and cone photoreceptors in mouse models of retinitis pigmentosa and diabetic retinopathy [21,22,23,37]. Yet, its effect in an AMD-related model is still unclear. Herein we determined whether S1R- or S2R-selective pharmacological modulation could protect photoreceptors in an AMD-related model of illumination-induced retinal degeneration in *Abca4*−/−*Rdh8*−/− mice. We found that pretreatment of the mice with either the S1R-selective drug (+)-pentazocine or the S2R-selective ligand CM398 mitigated photoreceptor loss.

Although studies have repeatedly shown the benefits of S1R agonists in protecting photoreceptors and ganglion cells [15,16], these effects on retinal neurons may not be extrapolatable to AMD pathology that intimately involves the RPE, a highly specialized non-neuronal tissue [26]. AMD is a complex and multifactorial chronic disease, and there has been a lack of mouse models to ideally recapitulate its pathological processes. A promising AMD-related model was recently established based on a disrupted visual cycle in *Abca4*−/−*Rdh8*−/− mice [27], in which illuminated retinas develop some key features of the dry form of clinical AMD and Stargardt disease [38]. These include lipofuscin and drusen deposition, Bruch’s membrane thickening, increase of pro-inflammatory cytokines and subretinal microglia and macrophages, and ultimately, RPE and photoreceptor atrophy [28,39,40]. Consistent with the photoreceptor-protective effect of S1R activation with (+)-pentazocine in illuminated *Abca4*−/−*Rdh8*−/− mice, S1R ablation worsened photoreceptor loss in oxidant-challenged mice. Thus, our data obtained from both AMD-related models support a specific role for S1R in photoreceptor protection. Another line of consistent evidence is that the S1R agonist SA4503 (cutamesine) mitigated photoreceptor cell death induced by light in wild-type mice [41] which, however, is not considered an AMD-associated model. Thus, to our knowledge, the current study represents the first to use AMD-related preclinical models to explore the potential of selective S1R or S2R modulation for photoreceptor protection in vivo.

Furthermore, using an in vitro model [42] (Figure S1), we found that pretreatment of ARPE19 cells with CM398 or (+)-pentazocine was protective for cell survival against paraquat-induced cytotoxicity, whereas PB28, which targets both S1R and S2R [43] did not show a protective effect. This result is consistent with the in vivo evidence that CM398 inhibits retinal autofluorescence (Figure 2), which is thought to be mainly emitted by the cytotoxic wastes accumulated in the RPE [40,44]. In addition, in a meeting abstract (https://iovs.arvojournals.org/article.aspx?articleid=2781135, accessed on 2 November 2022), S2R modulators (albeit unnamed) were described to improve photoreceptor outer segment trafficking in cultured ARPE-19 cells. Therefore, these studies underscore the importance of further investigating the role of S1R and S2R in the RPE via pharmacological modulation or genetic manipulation—an area that has thus far been underexplored [17,45,46].

Interestingly, the S2R-selective ligand CM398 appeared to largely rescue light-induced photoreceptor loss in *Abca4*−/−*Rdh8*−/− mice, as observed herein. Our previous study showed that TMEM97/S2R ablation exacerbated retinal degeneration in sodium iodate-treated mice [17]. While its definition as an S2R agonist or antagonist remains unclear [47], CM398 is characterized as a putative antagonist based on an animal behavior assay [48]. In this light, our current pharmacological study using CM398 and a previous genetic study [17] using *Tmem97*−/− mice appear paradoxical. It is possible that this discrepancy arose from the different retinal degeneration models used in these studies.

On the other hand, discrepant results from S2R pharmacological modulation and TMEM97 ablation have been recently observed by other investigators. For example, whereas anti-cancer properties have been traditionally attributed to putative S2R agonists [43,49,50], tumor growth was suppressed by TMEM97/S2R knockout in a breast cancer xenograft model [51]. Furthermore, a recent report from the Mach group suggested that TMEM97 does not mediate S2R ligand-induced cytotoxicity of HeLa cells [52]. In addition, while either the S2R-selective antagonist CM398 or S2R agonists showed anti-neuropathic pain effects [48,53], TMEM97/S2R ablation in mice did not lead to significant alterations in the pathogenesis of neuropathic pain [54]. Therefore, these studies implicate that using an antagonist to block the S2R binding site and eliminating the TMEM97 protein (knockout) may lead to different functional outcomes. This intriguing conundrum warrants future research to resolve. It is plausible to speculate that S2R/TMEM97 may function in ligand binding dependent or independent contexts under different circumstances.

There are limitations to this study. For example, we have obtained indirect evidence from retinal autofluorescence suggesting a protective effect of CM398 on the RPE. However, as the RPE and photoreceptors form a structurally and functionally integral unit, it is challenging to tease apart the disease processes in these tissues. More sophisticated determinations, such as two-photon microscopy and analysis with RPE whole mounts [44], are needed to gain detailed information on RPE pathophysiology, and the downstream signaling mechanisms remain to be elucidated. Moreover, since retinal degeneration is acutely induced by strong illumination in *Abca4*−/−*Rdh8*−/− mice, it would be interesting to re-examine the effect of (+)-pentazocine and CM398 in aged *Abca4*−/−*Rdh8*−/− mice that develop some chronic features of dry AMD even without retinal illumination [27,40]. In doing so, it is necessary to apply multiple approaches of determination including ERG, OCT, and histology using a greater number of animals for better statistical assessment. In addition, given our recent observations regarding S1R and S2R-associated sexual dimorphism [31], it would also be necessary to perform S1R and S2R modulations in female mice.

## 4. Conclusions

AMD is the primary cause of blindness in developed countries. Current anti-neovascular therapy is effective for late wet AMD but is associated with significant risks and is only applicable to ~10% of AMD patients. For the majority of the patients who have dry AMD, there is no approved clinical treatment available [26]. Herein, using a dry AMD-related model, we present evidence of photoreceptor protection afforded by (+)-pentazocine and CM398, the ligands highly selective to S1R and S2R, respectively. This study thus confers a compelling rationale for more research to evaluate S1R and S2R as potential therapeutic targets for treating dry AMD.

## 5. Methods

### 5.1. Ethics Statement

All animal procedures, including euthanization, conformed with the NIH Guide for the Care and Use of Laboratory Animals and complied with the Animal Care and Use Committees at the University of Wisconsin-Madison and the University of Virginia. Mice were reared on a normal diet with standard 12 h/12 h light/dark cycles unless otherwise specified. Animals were euthanized in a chamber gradually filled with CO_2_.

### 5.2. Abca4−/−Rdh8−/− Mice and the Retinal Illumination Model

Dr. Krzysztof Palczewski kindly provided us the double knockout mouse line in a C57BL/6 J × 129S1/SvImJ mixed background^27^. ERG and OCT experiments were performed at the University of Wisconsin-Madison, in 2015 (OCT, dated on the fundus photograph in Figure 3A) or 2016 (ERG). Genotype-confirmed male *Abca4*−/−*Rdh8*−/− mice at 8–10 weeks of age were dark-adapted overnight in a room equipped with dim (<5 lux) red light (12 h/12 h light/dark) and were injected i.p. with (+)-pentazocine (1 mg/kg), CM398 (3 mg/kg), or vehicle (DMSO of equivalent volume) 30 min before retinal illumination (10,000 lux for 30 min) [27], which was conducted in a ventilated temperature-controlled chamber equipped with 6 equally spaced ring lights. The mice were then returned to the room with dim red light and housed until post-illumination day 7, when ERG or OCT was performed.

### 5.3. Electron Retinography (ERG)

On post-illumination day 7, ERG was performed, and the animal was then euthanized for eye enucleation and histological analysis. To record the ERGs, we followed standard rodent procedures as stated in our previous reports [55,56]. Briefly, dark-adapted, anesthetized, and pupil-dilated mice were placed on a heated platform. ERG was recorded using the Celeris Ophthalmic Electrophysiology System (Diagnosys LLC, Lowell, MA, USA). Dark-adapted rod function was assessed at increasing flash intensities (0.03–30 cd·s/m^2^); c-waves were recorded using flashes of 2.5 and 25 cd·s/m^2^. All the ERG measurements were performed between 5 AM and 1 PM.

### 5.4. Sigmar1−/− Mice and NaIO_3_-Induced Retinal Degeneration Model

The *Sigmar1*−/− mouse colony was previously established [30]. Briefly, a heterozygous Oprs1 (Sigmar1) mutant line [OprsGt (IRESBetageo) 33Lex] in a C57BL/6 J × 129 s/SvEv mixed background was purchased from the Mutant Mouse Regional Resource Center (MMRRC, UC, Davis, CA, USA). A relatively uniform background was reached through back-crossing to C57BL/6 J mice (JAX#000664). This was occasionally repeated to refresh the colony and prevent genetic shift. Genotyping was frequently performed. Littermate *Sigmar1*+/+ mice were used as wild-type controls. To induce retinal degeneration, we used the established model of systemic delivery of the oxidant NaIO_3_ [32,57]. Male *Sigmar1*−/− mice and *Sigmar1*+/+ mice (8 weeks old) were anesthetized with isoflurane through inhalation (flow rate 2 mL/min), and received a single tail-vein injection of NaIO_3_ (30 mg/kg of body weight) or PBS control [17]. These experiments were performed at the University of Virginia.

### 5.5. Cryosection Preparation, Histology, and Measurement of Retinal ONL Thickness

The histological analyses were performed following the protocol in our recent report [17]. Briefly, mice were euthanized 3 days after NaIO_3_ injection. Enucleated eyes were fixed at 4 °C overnight in 4% paraformaldehyde. Cryosections were cut from frozen eyeballs and stained with hematoxylin and eosin (H&E). ONL thickness was measured by ImageJ in a blinded fashion. On each retinal section, three regions were chosen (0–1000 μm, 1000–2000 μm, or >2000 μm from the optic nerve head). The values from 3 or 4 sections (each including 3 regions) were averaged for each eye, and the means from total animals in the NaIO_3_- or PBS-treated groups were then averaged to calculate the mean and standard error of the mean (SEM).

### 5.6. Spectral-Domain Optical Coherence Tomography (SD-OCT)

On post-illumination day 7, SD-OCT data were collected following published methods [55,58]. Fundus photography data were obtained using the TRC 50EX retinal camera (Topcon Corp., Tokyo, Japan). SD-OCT data were collected using the Heidelberg™ Spectralis HRA + OCT instrument (Heidelberg Engineering, Heidelberg, Germany) and were analyzed using the accompanying software. Imaging included averaged volume intensity scans. The ONL thickness and the number of autofluorescent granules were determined by ImageJ. After OCT measurements, the animals were euthanized for eye enucleation and retinal sections.

## Supplementary Materials

The following supporting information can be downloaded at: https://www.mdpi.com/article/10.3390/genes13122386/s1, Figure S1: (+)-pentazocine and CM398 protect ARPE-19 cells against paraquat-induced cytotoxicity. Primary RPE cells were isolated from porcine eyes [59].
