# Supplementary material for: Retinal Photoreceptor Protection in an AMD-Related Mouse Model by Selective Sigma-1 or Sigma-2 Receptor Modulation
Source: Genes (Basel). 2022 Dec 16;13(12):2386. doi: 10.3390/genes13122386 (PMC9778362; doi:10.3390/genes13122386)
Supplement: Supplementary file 1 [file genes-13-02386-s001.zip › genes-2042836-supplementary.pdf]

genes

MDPI

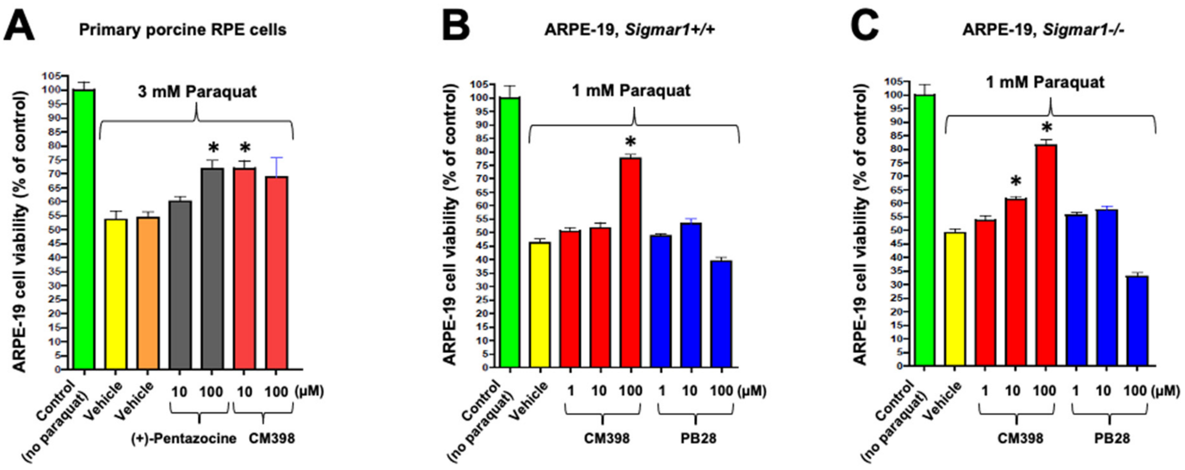

Figure S1. (+)-pentazocine and CM398 protect ARPE-19 cells against paraquat-induced cytotoxicity. Primary RPE cells were isolated from porcine eyes. The brown bar is an additional control, and the brown and yellow bars are the same vehicle condition. (**B** and **C**). ARPE-19 cells were purchased from American Type Culture Collection (ATCC, Manassas, VA). The S1R knockout ARPE-19 cell line was generated using the CRISPR-Cas9 gene editing approach [55]. Cells were cultured to ~80% confluency. Before paraquat treatment, the cell culture was treated with (+)-pentazocine, CM398, or PB28 (antagonist for S1R and putative agonist for S2R) to the indicated concentrations or vehicle control (equal amount of DMSO). The cells were treated with 3 mM ((**A**), primary porcine RPE) or 1 mM ((**B**), ARPE-19) paraquat for 24 h and then used for MTT assay to measure cell viability. Quantification: Mean ± SD, $n = 3$, one-way ANOVA with Bonferroni test, * $p < 0.05$ (compared to the basal condition represented by the yellow bar).
